# BRAF L485–P490 deletion mutant metastatic melanoma sensitive to BRAF and MEK inhibition: A case report and literature review

**DOI:** 10.3389/fphar.2022.1019217

**Published:** 2023-01-06

**Authors:** Simeng Zhang, Zichang Yang, Yu Cheng, Xiaoyu Guo, Chang Liu, Shuo Wang, Lingyun Zhang

**Affiliations:** ^1^ Department of Medical Oncology, The First Hospital of China Medical University, Shenyang, China; ^2^ Key Laboratory of Anticancer Drugs and Biotherapy of Liaoning Province, The First Hospital of China Medical University, Shenyang, China; ^3^ Liaoning Province Clinical Research Center for Cancer, Shenyang, China

**Keywords:** metastatic melanoma, BRAF in-frame deletion mutation, dabrafenib, trametinib, case report

## Abstract

**Background:** The combination therapy of BRAF inhibitors (BRAFis) and MEK inhibitors (MEKis) has been approved as a first-line treatment for metastatic melanoma with BRAF V600 mutants. Recently, BRAF mutations have been divided into three subtypes based on biochemical and signaling characteristics. Unlike V600 mutants that show class I BRAF mutations, evidence of the effects of using BRAF inhibitors and MEK inhibitors in patients with non-V600 BRAF mutations remains unclear. The exploration of effective therapy for non-V600 BRAF mutations in melanoma has thus attracted much interest.

**Case presentation:** We reported a case of a 64-year-old female metastatic melanoma patient with a novel BRAF *p*.L485–P490 deletion mutation. The patient received anti-PD1 agent pembrolizumab (100 mg) therapy as the first-line treatment for two cycles, which was terminated due to an intolerable adverse effect. Considering the *p*.L485–P490 deletion mutation signal as an active dimer which is akin to a class II BRAF mutation, the patient underwent dabrafenib and trametinib combination therapy as a second-line treatment. After two cycles of combination treatment, the patient achieved a partial response confirmed by radiological examinations. At the last follow-up date, the patient had obtained over 18 months of progression-free survival, and the treatment was well tolerated.

**Conclusion:** The combination therapy of dabrafenib and trametinib has been proven to be an effective method as a later-line therapy for metastatic melanoma patients with class II BRAF in-frame deletion mutations.

## 1 Introduction

In recent years, the mortality rate of melanoma has decreased by 7% annually due to the development of targeted therapies (Siegel et al., 2020). Approximately 40%–50% of melanoma patients have presented mutations in BRAF, and the substitution of valine with glutamic acid (termed as the V600E mutations) is reported in about 80% of melanoma patients with BRAF mutations (Davies et al., 2002). Currently, targeted therapies, including immune checkpoint inhibitors, BRAF inhibition therapy, and MEK inhibition therapy, have been approved for use as a first-line treatment in metastatic melanoma patients who carry BRAF oncogenic mutations (Bai and Flaherty, 2021). In contrast, non-V600 mutations are considered to be rare variants of BRAF which are present in 5%–15% of the population of melanoma (Dankner et al., 2018a). Although growing evidence has verified the benefit outcomes obtained from the combination therapy of BRAF inhibitors (BRAFis) and MEK inhibitors (MEKis) in BRAF V600 mutations (Long et al., 2014), however, the efficacy of BRAFis and MEKis in non-V600 mutation melanoma remains unclear. Nikanjam et al. found that patients with non-V600 E/K mutation presented poorer prognosis than the standard BRAF variants; nonetheless, 63% of non-V600E/K-mutation melanoma patients also obtained a clinical response (Nikanjam et al., 2021). Recently, BRAF mutations were divided into three subtypes based on three important biochemicals and signaling aspects of these mutants (Yao et al., 2015; Dankner et al., 2018a). Class I mutations refer to BRAF V600 variants, which act as RAS-independent monomeric substances. Non-V600 mutations denote class II and III subtypes with high and low kinase activities, respectively (Yao et al., 2017). Johnson DB et al. found that some class II BRAF mutations could benefit from BRAFi and/or MEKi treatment, and class III mutations showed limited response to the aforementioned therapies (Johnson and Dahlman, 2018).

In this case study, we report a class II BRAF rare mutant metastatic melanoma patient who has benefited from the BRAFi and MEKi combination therapy. The patients obtained progression-free survival (PFS) for 18 months at the last follow-up in March 2022.

## 2 Case presentation

**FIGURE 1:**
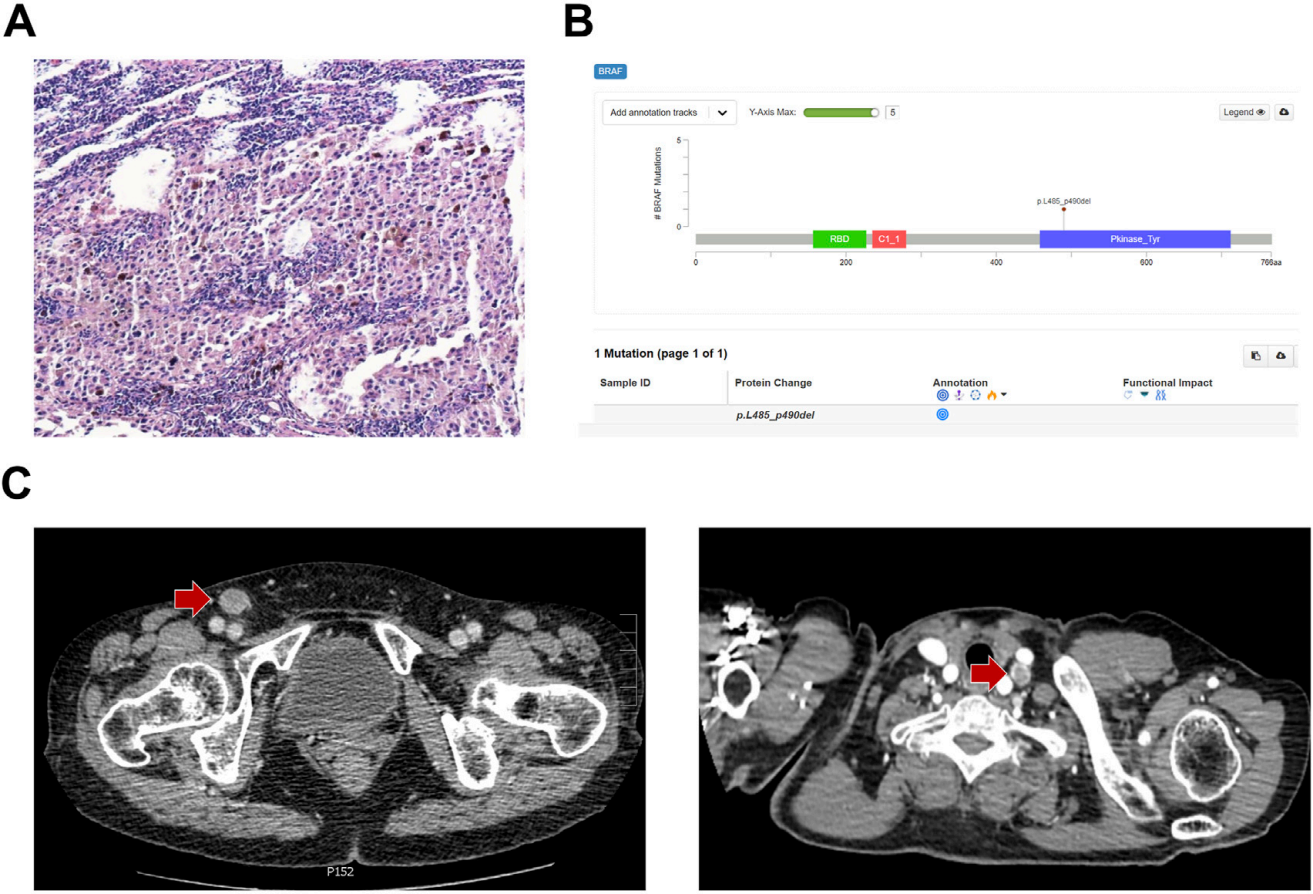
Melanoma history and gene status of the patient at baseline. **(A)** Immunohistochemistry and **(B)** next-generation sequencing results of the inguinal region mass biopsy. **(C)** Contrast-enhanced CT showed enhanced lymph nodes in the right inguinal region and the left cervical region at baseline.

A 64-year-old female patient who underwent laser therapy because of plantar melanocytic nevus in September 2017 was assessed; after 2 years, in October 2019, an isolated enlarged mass presented in the inguinal region and the pathological biopsy by surgical resection demonstrated lymph node metastatic melanoma (Figure 1A). PD-L1 [programmed cell death ligand 1: the tumor proportion score (TPS) of 60% and combined positive score (CPS) of 65%] and low tumor mutational burden (TMB) could be determined by the next-generation sequencing analysis. In addition, microsatellite stability and *p*.L485–P490 deletion mutation of BRAF were also found in this patient (Figure 1B). No other intervention was conducted after resection. In June 2020, an enhanced enlarged lymph node in the right inguinal region and the left cervical region was detected by contrast-enhanced computed tomography (CT), which indicated melanoma metastases (Figure 1C). Lactate dehydrogenase (LDH) of the patient was within normal limits (179 U/L), and the clinical stage was rcT0N0M1a based on the American Joint Committee on Cancer (AJCC) version 8.0.

**FIGURE 2:**
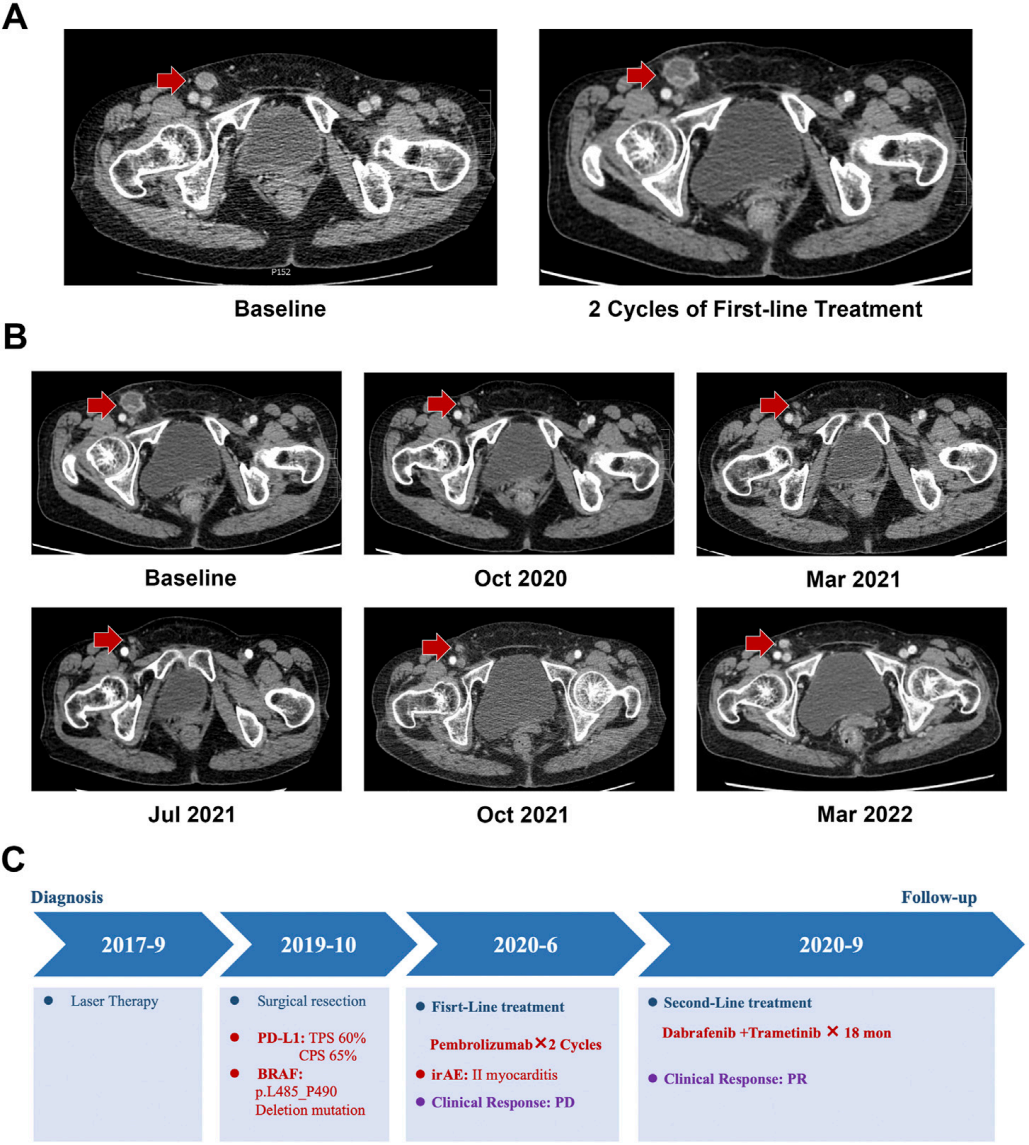
Medical imaging examination during the treatment period. **(A)** Progression disease (PD) confirmed by CT after two cycles of the first-line treatment. **(B)** Contrast-enhanced abdominal CT showed that the target lesion presented partial response (PR) to the dabrafenib and trametinib combination treatment. **(C)** Treatment summary of the metastatic melanoma patient with BRAF *p*.L485–P490 deletion mutations.

The patient underwent two cycles of anti-programmed cell death 1 (PD-1) with the antibody pembrolizumab (100 mg) as the first-line treatment (Figure 2A). After the second infusion of pembrolizumab, the treatment was stopped because of immune-related adverse events in the form of grade II myocarditis. In September 2020, after recovery from myocarditis, the target lesion located in the right inguinal region of the patient was found to be progressed by contrast-enhanced abdominal CT with the PFS for 2.5 months. The patient presented *p*.L485–P490 deletion mutation of BRAF which has only been mentioned in the cell lines of pancreatic and ovarian cancers. This novel BRAF in-frame deletion was classified as a class II BRAF mutation that might benefit from the BRAFi and MEKi combination therapy (Chen et al., 2016; Johnson and Dahlman, 2018). Thus, the patient underwent the combination therapy of dabrafenib (150 mg BID) and trametinib (2 mg QD) as a second-line treatment (Figure 2B). After two cycles of the combination therapy, significant regression of the target lesion in the inguinal region exhibited a partial response, as evinced by contrast-enhanced CT. The patient perceived that the symptoms of fatigue and inguinal region discomfort were significantly relieved. Until the last follow-up in March 2022, the PFS of the patient was over 18 months, and the target lesion still showed a partial response to the combination therapy (Figure 2C). The patient tolerated the combination therapy well, and only grade I fatigue was noted.

## 3 Discussion

Research indicated that melanoma patients who exhibited class I BRAF mutations could benefit from BRAFi or MEKi therapy. By comparison, class II mutations refer to non-V600 mutants that act as RAS-independent activated dimers which have intermediate kinase activities in activating the mitogen-activated protein kinase (MAPK) pathway compared to oncogenic RAS and BRAF-V600E/D mutants (Wan et al., 2004). Based on the location, class II mutations were divided into two subgroups located in the activation segment or P-loop. Commonly reported sites of BRAF class II mutations were L597/K601 in the activation segment and G466/G469 in the P-loop (Dankner et al., 2018a; Johnson and Dahlman, 2018). Class III mutations (G446/N851/D594/G596) show impaired kinase activity and increased MAPK signaling by wild-type RAF heterodimers (Wan et al., 2004; Yao et al., 2017). In this study, we reported a metastatic melanoma patient who presented BRAF *p*.L485–P490 deletion mutation.

The L485–P490 deletion mutation was first detected in the cell lines of the pancreatic, lung, and ovarian cancers, which is a novel BRAF in-frame deletion near the αC-helix region of the kinase domain and functions as a BRAF homodimer. Because the deletion and these mutant signals act as active dimers, L485–P490 deletion mutation was classified as a class II BRAF mutation (Yaeger and Corcoran, 2019; Song et al., 2022). In addition, the MAPK activation mediated by L485–P490 deletion mutation was resistant to RAF monomer inhibitors, such as vemurafenib, but sensitive to an RAF dimer inhibitor (Chen et al., 2016). Such a phenomenon was attributed to the fact that RAF inhibitors are preferred to bind to the inactive αC-out conformation instead of the active αC-in conformation of these mutations (Foster et al., 2016; Karoulia et al., 2016). As a rare class II BRAF mutation, there is insufficient evidence to select subsequent treatment for metastatic melanoma patients beyond first-line anti-PD-1 therapy. Kim et al. discovered that MEKis could induce a remarkable response to melanoma in patients with non-V600 BRAF mutations (Kim et al., 2013); however, due to the limited class II mutation frequency in melanoma, clinical trials which focus on the evaluation of the efficacy of a single agent BRAFi or MEKi in treating such a population remain rare. A recent phase II study evaluated the efficacy and safety of trametinib in advanced melanoma patients with non-V600 mutations. The result showed that the tumor objective response rate was 33%, median PFS was 7.3 months, and most responses were detected in class II mutations (22%), suggesting trametinib might be a choice for non-V600 BRAF mutant melanoma patients (Nebhan et al., 2021). Based on both *in vitro* and *in vivo* models, Dankner et al. found that class II BRAF mutations could benefit from a BRAFi and MEKi combination treatment rather than using a single MAPK inhibitor. In addition, researchers also reported two melanoma patient-derived xenografts harboring the BRAF L597 mutations obtained a temporary response and control of metastases from dabrafenib and trametinib (Dankner et al., 2018b). Although there is no direct evidence in treating melanoma with BRAF L485–P490 deletion mutations, which is analogous to class II mutants, the combination of BRAFis and MEKis may be an optimal therapy. Surprisingly, the patient achieved over 18 months of PFS, which was far more than the median PFS (3–7 months) of previous studies on BRAFi and MEKi therapy in treating non-V600 BRAF mutation melanoma (Menzer et al., 2019; Nebhan et al., 2021). These results suggest that combination therapy was a potential treatment strategy for melanoma patients with BRAF in-frame deletion mutations.

## 4 Conclusion

In conclusion, we presented a case of a metastatic melanoma patient with a novel L485–P490 deletion mutation who underwent dabrafenib and trametinib combination therapy as a second-line treatment and obtained over 12 months’ clinical response. The BRAFi and MEKi combination therapy might be an efficacious method for melanoma patients with rare deletion mutations of the BRAF oncogene. Indeed, this single case is not enough to reflect the landscape of BRAF deletion mutant melanoma, and further investigation by clinical trials is necessary. This study may provide new insights into treating metastatic melanoma patients with rare class II BRAF mutations.

## Data Availability

The original contributions presented in the study are included in the article/supplementary material; further inquiries can be directed to the corresponding authors.

## References

[B1] Bai X. Flaherty K. T. (2021). Targeted and immunotherapies in BRAF mutant melanoma: Where we stand and what to expect. Br. J. dermatology 185 (2), 253–262. 10.1111/bjd.19394 32652567

[B2] Chen S. H. Zhang Y. Van Horn R. D. Yin T. Buchanan S. Yadav V. (2016). Oncogenic BRAF deletions that function as homodimers and are sensitive to inhibition by RAF dimer inhibitor LY3009120. Cancer Discov. 6 (3), 300–315. 10.1158/2159-8290.CD-15-0896 26732095

[B3] Dankner M. Lajoie M. Moldoveanu D. Nguyen T. T. Savage P. Rajkumar S. (2018). Dual MAPK inhibition is an effective therapeutic strategy for a subset of class II BRAF mutant melanomas. Clin. cancer Res. official J. Am. Assoc. Cancer Res. 24 (24), 6483–6494. 10.1158/1078-0432.CCR-17-3384 29903896

[B4] Dankner M. Rose A. A. N. Rajkumar S. Siegel P. M. Watson I. R. (2018). Classifying BRAF alterations in cancer: New rational therapeutic strategies for actionable mutations. Oncogene 37 (24), 3183–3199. 10.1038/s41388-018-0171-x 29540830

[B5] Davies H. Bignell G. R. Cox C. Stephens P. Edkins S. Clegg S. (2002). Mutations of the BRAF gene in human cancer. Nature 417 (6892), 949–954. 10.1038/nature00766 12068308

[B6] Foster S. A. Whalen D. M. Özen A. Wongchenko M. J. Yin J. Yen I. (2016). Activation mechanism of oncogenic deletion mutations in BRAF, EGFR, and HER2. Cancer Cell. 29 (4), 477–493. 10.1016/j.ccell.2016.02.010 26996308

[B7] Johnson D. B. Dahlman K. B. (2018). Class matters: Sensitivity of BRAF-mutant melanoma to MAPK inhibition. Clin. cancer Res. official J. Am. Assoc. Cancer Res. 24 (24), 6107–6109. 10.1158/1078-0432.CCR-18-1795 PMC6295213 30042206

[B8] Karoulia Z. Wu Y. Ahmed T. A. Xin Q. Bollard J. Krepler C. (2016). An integrated model of RAF inhibitor action predicts inhibitor activity against oncogenic BRAF signaling. Cancer Cell. 30 (3), 501–503. 10.1016/j.ccell.2016.08.008 27622340

[B9] Kim K. B. Kefford R. Pavlick A. C. Infante J. R. Ribas A. Sosman J. A. (2013). Phase II study of the MEK1/MEK2 inhibitor Trametinib in patients with metastatic BRAF-mutant cutaneous melanoma previously treated with or without a BRAF inhibitor. J. Clin. Oncol. official J. Am. Soc. Clin. Oncol. 31 (4), 482–489. 10.1200/JCO.2012.43.5966 PMC4878037 23248257

[B10] Long G. V. Stroyakovskiy D. Gogas H. Levchenko E. de Braud F. Larkin J. (2014). Combined BRAF and MEK inhibition versus BRAF inhibition alone in melanoma. N. Engl. J. Med. 371 (20), 1877–1888. 10.1056/NEJMoa1406037 25265492

[B11] Menzer C. Menzies A. M. Carlino M. S. Reijers I. Groen E. J. Eigentler T. (2019). Targeted therapy in advanced melanoma with rare BRAF mutations. J. Clin. Oncol. official J. Am. Soc. Clin. Oncol. 37 (33), 3142–3151. 10.1200/JCO.19.00489 PMC10448865 31580757

[B12] Nebhan C. A. Johnson D. B. Sullivan R. J. Amaria R. N. Flaherty K. T. Sosman J. A. (2021). Efficacy and safety of trametinib in non-V600 BRAF mutant melanoma: A phase II study. Oncol. 26 (9), 731–e1498. 10.1002/onco.13795 PMC8417872 33861486

[B13] Nikanjam M. Tinajero J. Barkauskas D. A. Kurzrock R. (2021). BRAF V600E/V600K mutations versus nonstandard alterations: Prognostic implications and therapeutic outcomes. Mol. cancer Ther. 20 (6), 1072–1079. 10.1158/1535-7163.MCT-20-0861 33722853 PMC9264327

[B14] Siegel R. L. Miller K. D. Jemal A. (2020). Cancer statistics, 2020. CA a cancer J. Clin. 70 (1), 7–30. 10.3322/caac.21590 31912902

[B15] Song J. Kobayashi Y. Asano Y. Sato A. Taniguchi H. Ui-Tei K. (2022). Knockdown of 15-bp deletion-type v-raf murine sarcoma viral oncogene homolog B1 mRNA in pancreatic ductal adenocarcinoma cells repressed cell growth *in vitro* and tumor volume *in vivo* . Cancers (Basel) 14 (13), 3162. 10.3390/cancers14133162 35804932 PMC9264874

[B16] Wan P. T. Garnett M. J. Roe S. M. Lee S. Niculescu-Duvaz D. Good V. M. (2004). Mechanism of activation of the RAF-ERK signaling pathway by oncogenic mutations of B-RAF. Cell. 116 (6), 855–867. 10.1016/s0092-8674(04)00215-6 15035987

[B17] Yaeger R. Corcoran R. B. (2019). Targeting alterations in the RAF-MEK pathway. Cancer Discov. 9 (3), 329–341. 10.1158/2159-8290.CD-18-1321 30770389 PMC6397699

[B18] Yao Z. Torres N. M. Tao A. Gao Y. Luo L. Li Q. (2015). BRAF mutants evade ERK-dependent feedback by different mechanisms that determine their sensitivity to pharmacologic inhibition. Cancer Cell. 28 (3), 370–383. 10.1016/j.ccell.2015.08.001 26343582 PMC4894664

[B19] Yao Z. Yaeger R. Rodrik-Outmezguine V. S. Tao A. Torres N. M. Chang M. T. (2017). Tumours with class 3 BRAF mutants are sensitive to the inhibition of activated RAS. Nature 548 (7666), 234–238. 10.1038/nature23291 28783719 PMC5648058

